# Association of daily step counts and step intensity with mortality among US adults: a cross–sectional study of NHANES 2005–2006

**DOI:** 10.1186/s12876-025-03606-7

**Published:** 2025-01-20

**Authors:** Tianzhou Peng, Changhao Liu, Ti Yang, Leyi Liao, Qingping Li, Hanbiao Liang, Jiapeng Zhang, Chen Xie, Kai Wang, Chuanjiang Li

**Affiliations:** Division of Hepatobiliopancreatic Surgery, Department of General Surgery, Nanfang Hospital, Southern Medical University, 1838 North Guangzhou Avenue, Guangzhou, Guangdong 510515 China

**Keywords:** Step counts, Step intensity, All–cause mortality, Metabolic dysfunction–associated steatotic liver disease, Physical activity

## Abstract

**Background & aims:**

We aimed to describe the dose–response relationship between daily step counts and intensity with respect to all–cause mortality among US adults diagnosed with metabolic dysfunction–associated steatotic liver disease (MASLD).

**Methods:**

Using data from the National Health and Nutrition Examination Survey (NHANES) database spanning from 2005 to 2006, a cross–sectional study included 1,108 participants was performed to assess the relationship between daily step counts and step intensity with mortality.

**Results:**

A total of 1,108 participants from the NHANES study were included, with a mean age of 49.5 ± 0.9 years. The sample consisted of 533 (48.1%) women, 809(73%) non–Hispanic whites, 122 (10.8%) non–Hispanic blacks, 133 (12.0%) Hispanic, and 44 (4.2%) individuals of other racial backgrounds. Using multivariable–adjusted Cox proportional hazards models, we found that compared to participants in the light–step group, there was significantly lower risk of mortality in the moderate (hazard ratio [HR], 0.47 [95% CI, 0.32–0.69]), high (HR, 0.35 [95% CI, 0.21–0.61]) and vigorous (HR,0.45 [95% CI, 0.22–0.93]) step groups. Sensitivity and subgroup analyses confirmed that the association between step count and mortality remained robust. However, after adjusting for all covariates, greater step intensity was not significantly associated with lower mortality. Further analysis revealed that age, BMI, and self–rated health could have confounded the relationship between step intensity and survival, potentially obscuring any direct effect of step intensity on mortality.

**Conclusions:**

Accumulating a higher number of daily steps, rather than focusing on step intensity, was associated with a lower risk of all–cause mortality in individuals with MASLD. Our findings suggest that achieving 10,000 steps per day may be optimal for reducing the risk of all–cause mortality risk in this population.

**Supplementary Information:**

The online version contains supplementary material available at 10.1186/s12876-025-03606-7.

## Key points

*Question *How are the associations between daily step counts and step intensity with mortality among US adults with MASLD?

*Findings* In this cohort study of 1,108 adult participants diagnosed with MASLD, a curvilinear dose–response association was found between the number of daily step and a lower risk of all–cause mortality. However, after adjusting for all covariates, no significant association was found between step intensity and all–cause mortality.

*Meaning *The study’s findings suggest that walking 10,000 steps per day is identified as optimal for reducing all–cause mortality risk specifically among individuals with MASLD.

**Supplementary Information:**

The online version contains supplementary material available at 10.1186/s12876-025-03606-7.

## Introduction

Metabolic dysfunction–associated steatotic liver disease (MASLD), characterized by hepatic steatosis along with at least one cardiometabolic risk factor (CMRF) and no other known cause, is estimated to affect approximately 30% of adult worldwide [1, 2]. MASLD is not only a liver–related condition but also a multi–system disease associated with cardiovascular disease (CVD) and extrahepatic cancers [3, 4]. Previous studies have shown that MASLD is associated with an increased risk of all–cause mortality [5].Currently, no pharmacological treatments are approved specifically for MASLD, making lifestyle interventions, including physical activity, the primary approach for managing the disease [5, 6].

Among physical activities, walking is one of the most accessible and popular options for the general population [7]. Over the past decade, the use of consumer–grade wearable activity monitors has increased substantially. These devices provide real–time feedback on both step counts and step intensity, allowing individuals to track their physical activity more effectively. Several studies leveraging this technology have shown that step–based recommendations are key to reducing mortality risk, with evidence drawn from diverse populations, including US adults [8], older women [9] and young adults [10]. However, to date, research focused on individuals with MASLD remains limited. Given the high prevalence of MASLD, the prevalence of MASLD underscores the urgency of developing specialized, evidence–based guidelines for this population [11]. Despite the importance of physical activity, no public health guidelines currently specify the optimal number of steps MASLD patients should take daily to reduce their mortality risk. Moreover, walking can be performed at different intensity levels, adding complexity to assessing its effects on health outcomes [9, 12, 13]. However, the relationship between step intensity and mortality remains unclear.

The National Health and Nutrition Examination Survey (NHANES) (www.cdc.gov/nchs/nhanes) is designed to assess the health and nutritional status of adults and children in the United States [8]. The survey is distinctive in that it incorporates both physical examinations and interviews. Several cross–sectional, nationally representative health examination surveys are part of the NHANES program. Questions about demographics, health insurance status, dietary habits, acute and chronic medical issues, mental health, and prescription drug use are all included in the health interview. Exam components can change between survey cycles but typically include blood pressure, dental exams, vision, hearing, dermatology, fitness, balance and strength testing, respiratory testing, taste and smell, and body measurements (weight, height, skin folds, body composition scans). Hematology, organ and endocrine function (e.g., thyroid, kidney), environmental exposure, dietary biomarkers, metabolic and cardiovascular health, and infectious disease are some laboratory components. In this study, we present data from NHANES to examine the associations of daily step counts and step intensity with mortality in individuals with MASLD. Additionally, our secondary objective was to explore whether step intensity is independently associated with mortality.

## Methods

### Study population

**Fig. 1:**
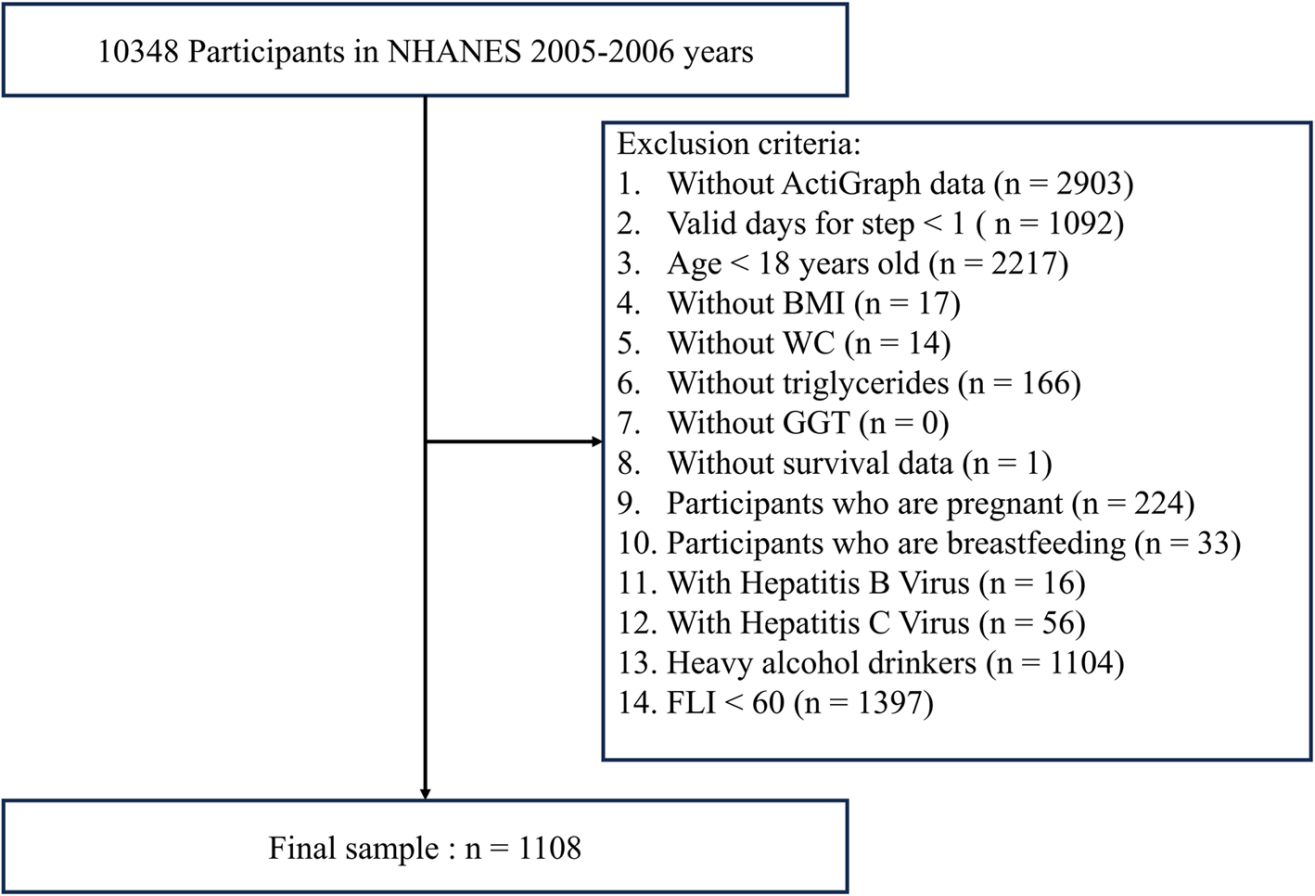
Flow chart of study inclusion legend: flowchart of the participants’ selection from NHANES 20005–2006. BMI, body mass index; FLI, fatty liver index; HDL–C, high–density lipoprotein cholesterol; GGT, Gamma Glutamyl Transpeptidase; NHANES, National Health, and Nutrition Examination Survey; WC, waist circumference

Data from 10,348 participants in the 2005–2006 NHANES cycle were linked to the National Death Index through 2019. As outlined in previous studies [1, 5], MASLD was defined based on the presence of hepatic steatosis in conjunction with at least one of the following five cardiometabolic risk factors: (1) body mass index (BMI) > 25 kg/m^2^ or waist circumference (WC) > 94 cm in men and > 80 in women [14]; (2) fasting serum glucose ≥ 100 mg/dL (≥ 5.6 mmol/L), 2–hour post–load glucose level ≥ 140 mg/dL (≥ 7.8 mmol/L), glycated hemoglobin (HbA1c) ≥ 5.7%, or on specific drug treatment [15]; (3) blood pressure ≥ 130/85 mmHg or on specific drug treatment [16]; (4) plasma triglycerides ≥ 150 mg/dL (≥ 1.70 mmol/L) or on specific drug treatment [17]; (5) plasma high–density lipoprotein cholesterol (HDL –C) < 40 mg/dL (< 1.0 mmol/L) for men and < 50 mg/dL (< 1.3 mmol/L) for women or on specific drug treatment [17]. Furthermore, the definition of MASLD continues to limit alcohol intake in the context of steatosis, with an average weekly intake of 140 g for women and 210 g for men [1]. The exclusion criteria were set forth as follows: (1) loss of ActiGraph data, (2) fewer than one valid day (with wearing time ≥ 10 h) for step counts data, (3) age below 18 years, (4) missing data for BMI, WC, triacylglycerol levels, gamma glutamyl transpeptidase (GGT) levels, or survival status, (5) pregnancy or breastfeeding, (6) presence of hepatitis B/C virus, (7) heavy drinking(weekly alcohol intake ≥ 140 g in women, ≥ 210 g in men), and (8) fatty liver index (FLI) ≤ 60. This cohort study was approved by the Institutional Review Board (IRB) at each participating field center [18]. The flowchart detailing participant selection from the 2005 to 2006 NHANES cycle is illustrated (Fig. 1). All participants provided written informed consent, and the final cohort included 1,108 participants.

### Stepping amount and intensity

During the 2005-2006 NHANES cycle, participants wore an accelerometer (ActiGraph, model 7164) at the waist for 7 consecutive days during waking hours [19, 20]. To assess weekly step consistency, we included only participants who wore the accelerometer for at least 10 h on one day with recorded step counts [8]. Total daily step counts were calculated by summing steps on valid days and determining the median for each participant. Participants were categorized into four groups based on their average daily steps: light (< 4,000 steps/day), moderate (4,000 to 7,999 steps/day), high (8,000 to 11,999 steps/day), and vigorous (≥ 12,000 steps/day) [8]. Additionally, we calculated peak 60-min (peak 60 cadences), peak 30-min (peak 30 cadences), and peak 1-min (peak 1 cadences) cadences [21]. For peak 60 cadences and peak 30 cadences, the average was calculated by selecting the highest 60 and 30 cadences values each day, respectively, computing their daily mean, and averaging across all valid days. For peak 1 cadences, the highest cadences value each day was selected, and its mean was calculated across all days.

### Mortality ascertainment

To ascertain participants’ vital status, participants or their designated proxies were contacted semi–annually; furthermore, systematic searches of the National Death Index were conducted on a 5–year cycle [22]. Staff requested death certificates, hospital records for deaths, and autopsy reports. For this analysis, participants were followed up for death through December 31, 2019.

### Statistical analysis

To account for the complex multi-stage cluster design, NHANES sample weights were applied. Categorical data are presented as numbers (percentages) and compared using chi-squared tests, while continuous variables are reported as means with standard errors (SE) and compared using Student’s t-tests.

After verifying the proportional hazards (PH) assumption, Cox proportional hazards regression models were used to calculate hazard ratios (HRs) and 95% confidence intervals (CIs) for the association between step volume groups and all–cause mortality. These analyses were sequentially refined through tiered model adjustments: Model 1 adjusted for basic variables such as age and gender; Model 2 was further refined by including race, poverty–income ratio, education, BMI, waist circumference (WC), marital status, smoking status, comorbidities, diet, and alcohol consumption; and Model 3 was augmented by integrating cardiometabolic risk factors—central obesity, impaired glucose metabolism, hypertension, HDL–C, and elevated triglycerides. Additionally, restricted cubic splines, with four knots strategically positioned at the 5th, 35th, 65th, and 95th percentiles of the daily step counts distribution, were used to examine the dose–response association with mortality. Cox proportional hazard model was then conducted to explore the relationship between step intensity and all-cause mortality using a similar modeling approach.

Several sensitivity analyses were conducted. First, to reduce reverse causality, participants who died within the first two years were excluded. Next, to assess the influence of self-reported general health, analyses were conducted for participants with MASLD, excluding those who rated their health as “fair” or “poor.” Finally, analyses were adjusted for subgroups based on age, gender, and race. Statistical significance was defined as a two–sided *P*–value below 0.05. All statistical analyses were performed using SAS version 9.4 (SAS Institute).

## Results

In the study, a total of 1,108 subjects were enrolled, and their initial baseline characteristics are presented (Table 1). The mean age at baseline was 49.5 years, with a standard deviation of 0.9 years. Of these individuals, 533 (48.1%) were women and 575 (51.9%) were men. Racially, 809 (73%) were non-Hispanic whites, 122 (10.8%) were non-Hispanic blacks, 133 (12.0%) were Hispanic, and 44 (4.2%) were from other racial backgrounds. The median follow–up duration for participants was 13.8 years. Participants with MASLD who engaged in higher levels of physical activity, as measured by step counts, were younger, had lower BMI and smaller waist circumferences, and were more likely to be cohabiting and identified as moderate drinkers. They also had a lower prevalence of cardiometabolic risk factors, such as hypertension and impaired glucose metabolism, and were less likely to rate their general health as fair or poor.

**Table 1:** Characteristics of steps per day of participants in the study

Baseline demographics	<4,000 (*n* = 127)	4,000–7,999 (*n* = 372)	8,000–11,999 (*n* = 386)	≥12,000 (*n* = 223)	Total (*N* = 1,108)	Test of significance
**Age, y, means (SE)**	63.9(1.6)	53.1(1.3)	46.0(1.0)	43.8(0.8)	49.5 (0.9)	< 0.001(T–test)
**Gender, n (%)**						
Male	52(41.2)	139(37.4)	205(53.2)	179(80.4)	575(51.9)	< 0.001(Chi–square test)
Female	75(58.8)	233(62.6)	181(46.8)	44(19.6)	533(48.1)	
**Race, n (%)**						
Non–Hispanic whites	96(76.1)	281(75.6)	284(73.6)	148(66.4)	809(73)	< 0.001(Chi–square test)
Non–Hispanic blacks	17(13.0)	41(11.1)	42(10.9)	22(9.8)	122(10.8)	
Hispanic	6(4.5)	33(8.8)	47(12.2)	45(20.3)	133(12.0)	
Other Races	8(6.4)	17(4.6)	13(3.3)	8(3.5)	44(4.2)	
**BMI, kg/m**^**2**^**, means (SE)**	36.7(1.0)	35.1(0.4)	33.9(0.5)	31.8(0.3)	34.1(0.3)	< 0.001(T–test)
**WC, cm, means (SE)**	118.5(1.8)	112.9(0.9)	110.6(1.2)	107.2(0.6)	111.4 (0.7)	< 0.001(T–test)
**Folate intake, mcg, means (SE)**	147.2(12.0)	174.2(8.4)	171.4(7.9)	173.7(9.6)	170.4(4.6)	0.0383(T–test)
**Low–carb and low–fat diets,n(%)**						
Yes	83(65.4)	224(60.2)	234(60.6)	112(50.2)	653(58.9)	0.020(Chi–square test)
No	44(34.6)	148(39.8)	152(39.4)	111(49.8)	455(41.1)	
**Marital status, n (%)**						
Cohabitation	59(46.5)	229(61.6)	265(68.7)	175(78.5)	728(65.7)	< 0.001(Chi–square test)
Solitude	68(53.5)	143(38.4)	121(31.3)	48(21.5)	380(34.3)	
**Educational level, n (%)**						
Less than high school	10(7.5)	20(5.4)	21(5.5)	16(7.3)	67(6.0)	0.102(Chi–square test)
High school	18(14.6)	32(8.6)	33(8.6)	32(14.2)	115(10.3)	
More than high school	99(77.9)	320(86)	332(85.9)	175(78.5)	926(83.7)	
**Poverty–income ratio, n (%)**						
<1.3	35(27.0)	50(13.5)	46(11.8)	33(14.6)	164(14.4)	0.010(Chi–square test)
1.3–3.5	56(44.3)	148(39.7)	135(35.0)	89(40.1)	428(38.4)	
>3.5	32(25.3)	161(43.2)	197(51.0)	98(44.1)	488(44.7)	
Unknown	4(3.3)	13(3.5)	8(2.1)	3(1.1)	28(2.5)	
**Cardiometabolic risk factor (yes), n (%)**						
Central obesity	127(100.0)	371(99.9)	378(99.0)	220(98.9)	1096(98.9)	0.407(Chi–square test)
Impaired glucose metabolism	88(69.3)	209(56.3)	183(47.5)	91(40.6)	571(51.5)	0.006(Chi–square test)
Hypertension	97(76.8)	247(66.5)	216(55.9)	95(42.7)	655(59.1)	< 0.001(Chi–square test)
Low HDL–C	95(75.1)	227(61.1)	230(59.6)	120(53.9)	672(60.6)	0.036(Chi–square test)
High triglycerides	101(79.3)	248(66.7)	259(67.2)	155(69.5)	763(68.9)	0.265(Chi–square test)
**Alcohol consumption, %**						
Never	72(56.4)	156(41.9)	112(29.1)	54(24.0)	394(34.8)	< 0.001(Chi–square test)
Moderate	55(43.6)	216(58.1)	274(70.9)	169(76.0)	714(65.2)	
**Smoking status, %**						
Never	62(49.2)	202(54.2)	224(58.1)	111(49.9)	599(54.3)	0.019(Chi–square test)
Former	45(35.1)	113(30.4)	94(24.3)	56(25.2)	308(27.4)	
Current	13(10.6)	31(8.4)	38(9.8)	23(10.1)	105(9.5)	
Unknown	7(5.2)	26(6.9)	30(7.9)	33(14.8)	96(8.7)	
**Self–rated health**						
Excellent	6(4.5)	17(4.6)	20(5.2)	20(8.8)	63(5.7)	0.001(Chi–square test)
Very good	18(14.2)	113(30.3)	131(33.9)	86(38.5)	348(31.8)	
Good	43(33.8)	154(41.4)	174(45.0)	93(41.9)	464(42.2)	
Fair or poor	37(29.0)	48(13.0)	29(7.5)	11(5.1)	125(10.8)	
Unknown	23(18.5)	40(10.6)	32(8.4)	13(5.7)	108(9.5)	
**Comorbidities**						
** Coronary heart disease**						
Yes	15(11.8)	22(5.9)	14(3.6)	3(1.3)	54(4.9)	< 0.001(Chi–square test)
No	111(87.4)	347(93.3)	372(96.4)	220(98.7)	1050(94.7)	
Unknown	1(0.8)	3(0.8)	0(0)	0	4(0.4)	
**Stroke**						
Yes	21(16.5)	13(3.5)	9(2.3)	2(0.9)	45(4.1)	< 0.001(Chi–square test)
No	105(82.7)	358(96.2)	377(97.7）	221(99.1)	1061(95.8)	
Unknown	1(0.8)	1(0.3)	0(0)	0(0)	2(0.1)	
**Diabetes mellitus**						
Yes	44(34.6)	62(16.7)	54(14.0)	13(5.8）	173(15.6)	< 0.001(Chi–square test)
No	76(59.8)	300(80.6)	318(82.4)	208(93.2)	902(81.4)	
Unknown	7(5.6)	10(2.7)	14(3.6)	2(1.0)	33(3.0)	

*BMI *Body mass index, *HDL–C *High–density lipoprotein cholesterol, *SE *Standard Error, *WC *Waist circumference

Data are presented as means and standard error (SE) for continuous variables, number and proportions for categorical variables

### Step counts and all–cause mortality

**Fig. 2:**
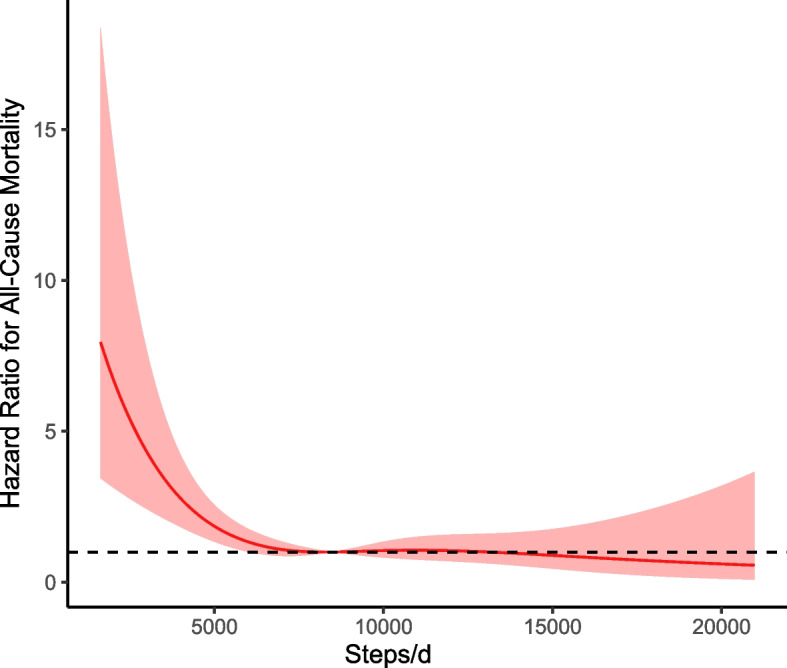
Restricted cubic spline (RCS) plots of adjusted dose–response relationships for steps perday and hazard ratio for all–cause mortality in participants with MASLD. The Restricted cubic spline (RCS) plots was adjusted for various factors, including age, gender, race, poverty–income ratio, education, BMI, WC, marital status, smoking status, alcohol consumption, self–rated health, complications(stroke, diabetes mellitus, coronary heart disease), diets, folate intake as well as components of the cardiometabolic risk factors such as central obesity, impaired glucose metabolism, hypertension, low HDL–C, and high triglycerides. BMI, body mass index; HDL–C, high–density lipoprotein cholesterol; HR, hazard ratio; MASLD, metabolic dysfunction–associated steatotic liver disease; WC, waist circumference

Preliminary analysis revealed a considerable attenuation in the association between daily step counts and mortality with incremental adjustment for various covariates (Table 2). Therefore, we present results from models that account for all these covariates. In the fully adjusted analysis, taking 4,000 to 7,999 steps per day was significantly associated with lower all-cause mortality compared to the reference group (< 4,000 steps/day) (HR, 0.47 [95% CI, 0.32–0.69]). Similarly, individuals taking 8,000 to 11,999 steps per day had a significantly lower mortality risk (HR, 0.35 [95% CI, 0.21–0.61]). Taking 12,000 steps or more per day was associated with a substantial reduction in all-cause mortality (HR, 0.45 [95% CI, 0.22–0.93]). Restricted cubic splines revealed a decline in mortality risk across the step-count distribution, leveling off around 10,000 steps per day (Fig. 2).

**Table 2:** Multivariable hazard ratios of steps per day and all–cause mortality in participants with MASLD

Steps/d	Model 1		Model 2		Model 3	
	HR (95% CI)	Wald Test	HR (95% CI)	Wald Test	HR (95% CI)	Wald Test
< 4,000	Reference		Reference		Reference	
4,000–7,999	0.39(0.28–0.53)	< 0.001	0.44(0.31–0.63)	< 0.001	0.47(0.32–0.69)	< 0.001
8,000–11,999	0.22(0.14–0.36)	< 0.001	0.35(0.21–0.58)	< 0.001	0.35(0.21–0.61)	< 0.001
≥ 12,000	0.24(0.09–0.64)	0.007	0.42(0.21–0.87)	0.018	0.45(0.22–0.93)	0.032

Model 1 was adjusted for age, gender; Model 2 was adjusted for model 1 + race, poverty–income ratio, education, BMI, WC, marital status, smoking status, alcohol consumption; self–rated health, complications(stroke, diabetes mellitus, coronary heart disease), diets, folate intake; Model 3 was adjusted for model 2 + components of the cardiometabolic risk factor (central obesity, impaired glucose metabolism, hypertension, low HDL–C and high triglycerides)

*BMI *Body mass index, *HDL–C *High–density lipoprotein cholesterol, *HR *Hazard ratio, *MASLD *Metabolic dysfunction–associated steatotic liver disease, *WC *Waist circumference

### Step intensity and all–cause mortality

**Fig. 3:**
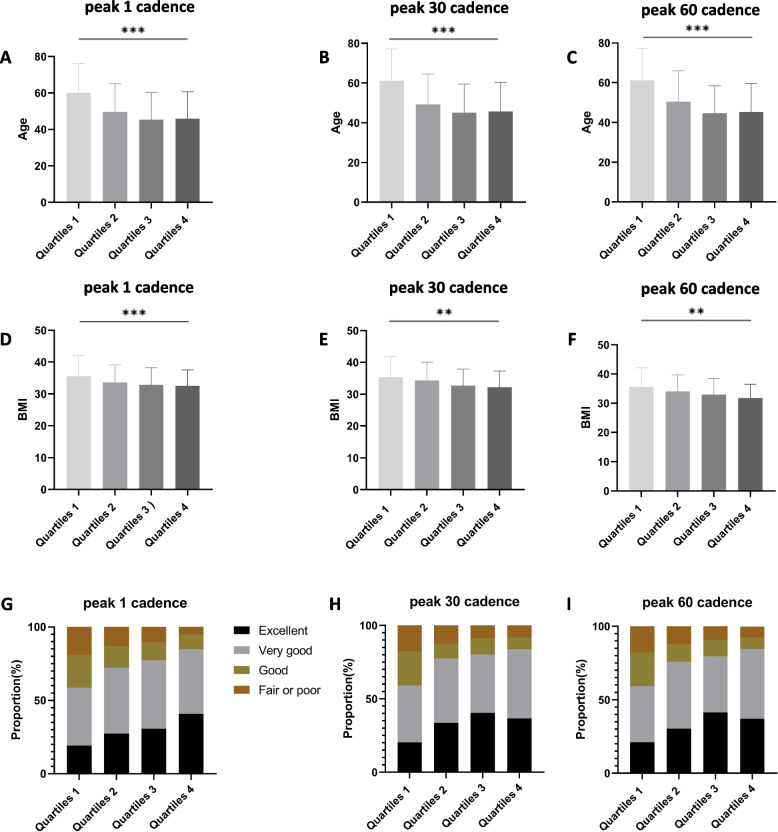
Histogram of Self–Rated Health Proportion, Age, and BMI in Different Step Intensity. BMI, body mass index; peak 60 cadences, peak 60–minute cadences; peak 30 cadences, peak 30–minute cadences; peak 1 cadences, peak 1–minute cadences

Our findings indicate that higher step intensity (78.0 to 107.0 steps per minute) was significantly associated with lower all-cause mortality for peak 1 cadences, compared to low step intensity (< 78.0 steps per minute), after adjusting for age and gender (Table 3). However, the highest step intensity group (> 107.0 steps per minute) did not show a significant reduction in mortality after similar adjustments (HR, 0.53 [95% CI, 0.23–1.24]). Cox proportional hazard model (Supplementary Table 1) revealed age as a significant confounder. Individuals with higher walking intensity tended to be younger, while older individuals were more likely to engage in lower-intensity activities (Fig. 3A). Mortality risk for peak 30 and peak 60 cadences decreased when adjusted for just age and gender but became statistically insignificant after adjusting for all covariates. After accounting for all confounding factors (Supplementary Tables 2–4), the association between step intensity and mortality was no longer statistically significant, primarily due to the older age, higher BMIs, and poorer health in individuals with lower step intensity (Fig. 3A–I).

**Table 3:** Multivariable hazard ratios of stepping intensity and all–cause mortality in participants with MASLD

Step intensity	Model 1		Model 2		Model 3	
Peak 1 cadences (steps per minute)	HR (95% CI)	Wald Test	HR (95% CI)	Wald Test	HR (95% CI)	Wald Test
Quartiles 1 (< 77.9)	Reference		Reference		Reference	
Quartiles 2 (78.0–96.5)	0.61(0.43–0.86)	0.008	0.75(0.50–1.12)	0.157	0.78(0.52–1.17)	0.222
Quartiles 3 (96.6–107.0)	0.44(0.24–0.82)	0.013	0.85(0.52–1.37)	0.497	0.84(0.52–1.38)	0.496
Quartiles 4 (≥ 107.1)	0.53(0.23–1.24)	0.133	0.65(0.34–1.26)	0.206	0.65(0.33–1.27)	0.210
Peak 30 cadences (steps per minute)						
Quartiles 1 (< 46.3)	Reference		Reference		Reference	
Quartiles 2 (46.4–64.2)	0.52(0.37–0.75)	0.001	0.73(0.48–1.12)	0.151	0.71(0.46–1.10)	0.129
Quartiles 3 (64.3–76.2)	0.42(0.27–0.65)	0.001	0.71(0.41–1.23)	0.223	0.73(0.42–1.28)	0.277
Quartiles 4 (≥ 76.3)	0.37(0.21–0.67)	0.003	0.71(0.38–1.32)	0.275	0.65(0.34–1.24)	0.195
Peak 60 cadences (steps per minute)						
Quartiles 1 (< 37.0)	Reference		Reference		Reference	
Quartiles 2 (37.1–52.4)	0.55(0.29–1.04)	0.064	0.76(0.50–1.14)	0.186	0.75(0.49–1.13)	0.171
Quartiles 3 (52.5–63.7)	0.34(0.19–0.6)	0.001	0.73(0.41–1.32)	0.302	0.77(0.42–1.40)	0.391
Quartiles 4 (≥ 63.8)	0.4(0.19–0.87)	0.024	0.65(0.34–1.25)	0.199	0.61(0.31–1.19)	0.144

Model 1 was adjusted for age, gender; Model 2 was adjusted for model 1 + race, poverty–income ratio, education, BMI, WC, marital status, smoking status, alcohol consumption, self–rated health, complications(stroke, diabetes mellitus, coronary heart disease), diets, folate intake; Model 3 was adjusted for model 2 + components of the cardiometabolic risk factor (central obesity, impaired glucose metabolism, hypertension, low HDL–C and high triglycerides)

*BMI *Body mass index, *HDL–C *High–density lipoprotein cholesterol, *HR *Hazard ratio, *MASLD *Metabolic dysfunction–associated steatotic liver disease, *WC *Waist circumference

### Sensitivity analysis

**Fig. 4:**
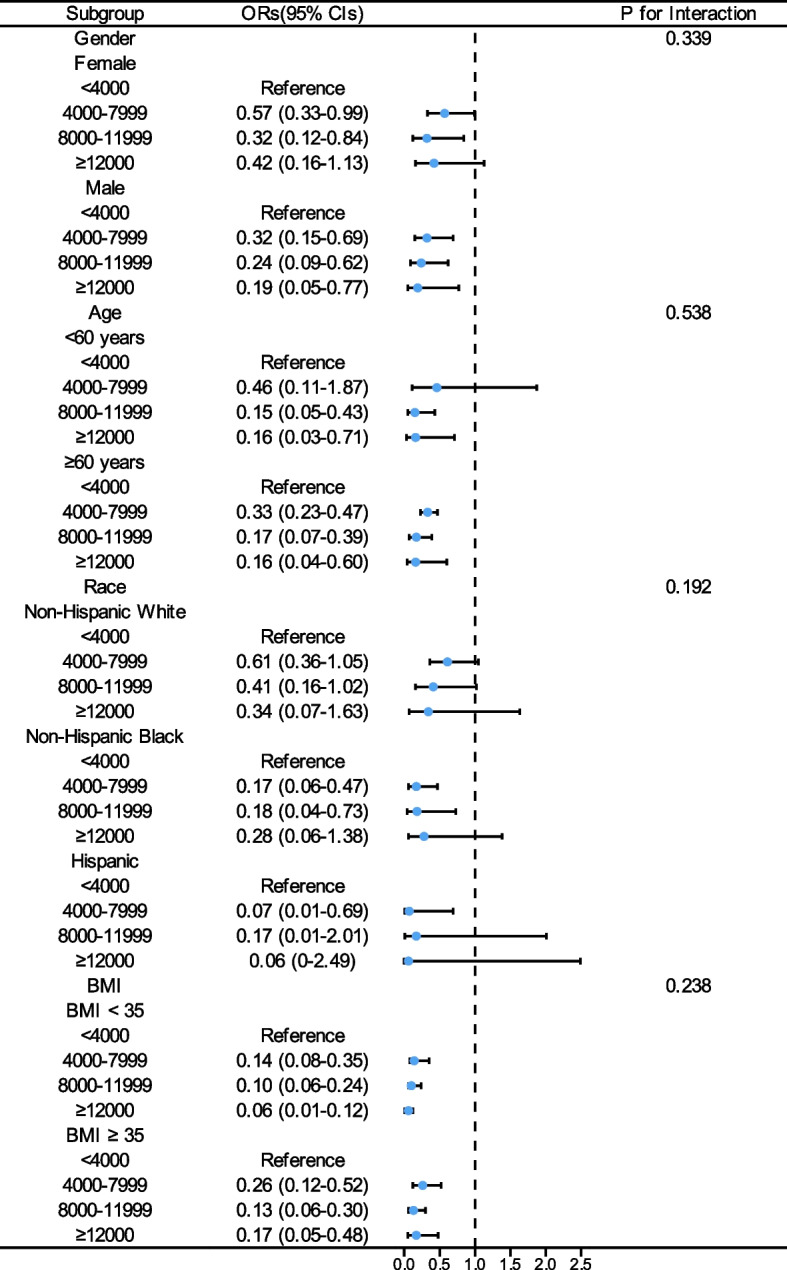
Forest Plot of the Association Between Daily Steps and All–Cause Mortality in MASLD Participants by Gender, Age, Race and BMI Subgroups. BMI, body mass index; OR, odds ratios; 95% CI, confidence intervals MASLD, metabolic dysfunction–associated steatotic liver disease

**Fig. 5:**
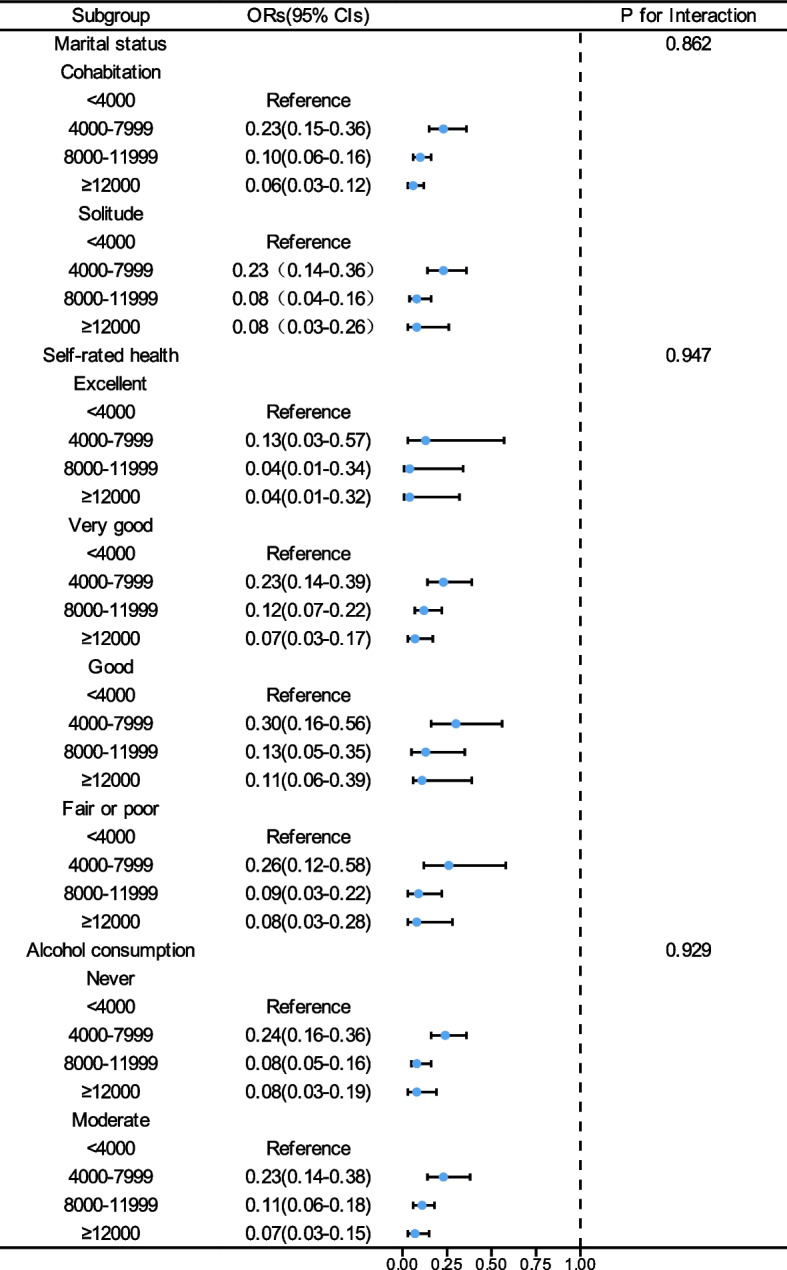
Forest Plot of the Association Between Daily Steps and All–Cause Mortality in MASLD Participants by Alcohol consumption, Marital status and Self–rated health Subgroups. OR, odds ratios; 95% CI, confidence intervals; MASLD, metabolic dysfunction–associated steatotic liver disease

**Fig. 6:**
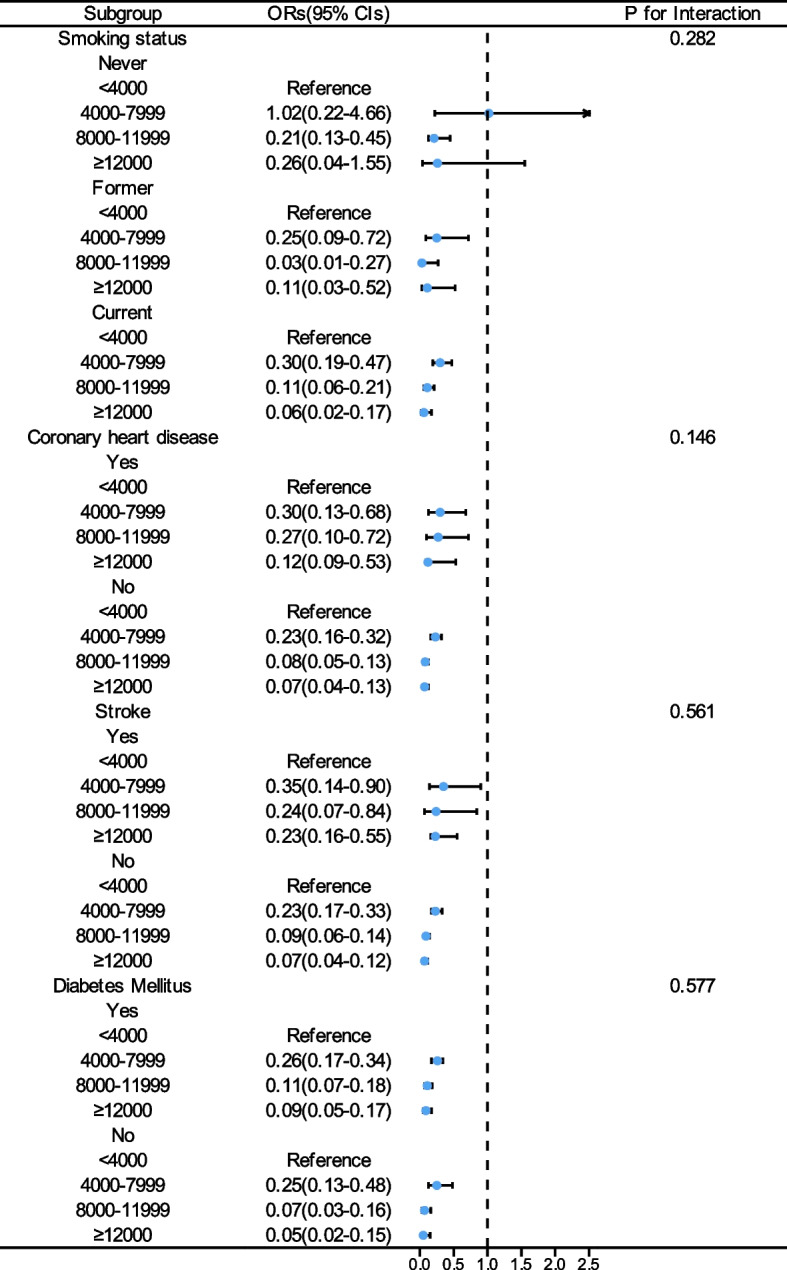
Forest Plot of the Association Between Daily Steps and All–Cause Mortality in MASLD Participants by Smoking status and Complications (Stroke, Diabetes mellitus, Coronary heart disease) Subgroups. OR, odds ratios; 95% CI, confidence intervals; MASLD, metabolic dysfunction–associated steatotic liver disease

Given the significant association we found between step counts and all–cause mortality, we conducted several sensitivity analyses to assess the robustness of our findings. The pattern remained consistent after excluding participants who died within the first two years (Table 4) and those who rated their health as fair or poor (Table 5). Subgroup analyses also showed no significant interactions between step count and variables such as age, gender, race, BMI, alcohol consumption, marital status, self-rated health, smoking status, coronary heart disease, stroke, and diabetes mellitus (Figs. 4, 5 and 6).

**Table 4:** Multivariable hazard ratios of steps per day and all–cause mortality in participants with MASLD excluding participants who died within the first 2 years of follow–up

Steps/d	Model 1		Model 2		Model 3	
	HR (95% CI)	Wald Test	HR (95% CI)	Wald Test	HR (95% CI)	Wald Test
< 4,000	Reference		Reference		Reference	
4,000–7,999	0.4(0.29–0.56)	< 0.001	0.46(0.32–0.67)	< 0.001	0.50(0.34–0.75)	< 0.001
8,000–11,999	0.21(0.13–0.35)	< 0.001	0.34(0.20–0.58)	< 0.001	0.36(0.21–0.62)	< 0.001
≥ 12,000	0.24(0.08–0.71)	0.013	0.44(0.21–0.92)	0.028	0.46(0.22–0.99)	0.047

Model 1 was adjusted for age, gender; Model 2 was adjusted for model 1 + race, poverty–income ratio, education, BMI, WC, marital status, smoking status, alcohol consumption, self–rated health, complications(stroke, diabetes mellitus, coronary heart disease), diets, folate intake; Model 3 was adjusted for model 2 + components of the cardiometabolic risk factor (central obesity, impaired glucose metabolism, hypertension, low HDL–C and high triglycerides)

*BMI *Body mass index, *HDL–C *High–density lipoprotein cholesterol, *HR *Hazard ratio, *MASLD *Metabolic dysfunction–associated steatotic liver disease, *WC *Waist circumference

**Table 5:** Multivariable hazard ratios of steps per day and all–cause mortality in participants with MASLD excluding participants self–reporting their health as fair/poor

Steps/d	Model 1		Model 2		Model 3	
	HR (95% CI)	Wald Test	HR (95% CI)	Wald Test	HR (95% CI)	Wald Test
< 4,000	Reference		Reference		Reference	
4,000–7,999	0.39(0.26–0.58)	< 0.001	0.43(0.30–0.61)	< 0.001	0.46(0.31–0.67)	< 0.001
8,000–11,999	0.22(0.13–0.37)	< 0.001	0.34(0.21–0.56)	< 0.001	0.34(0.20–0.58)	< 0.001
≥ 12,000	0.26(0.09–0.70)	0.011	0.40(0.20–0.81)	0.010	0.42(0.20–0.87)	0.019

Model 1 was adjusted for age, gender; Model 2 was adjusted for model 1 + race, poverty–income ratio, education, BMI, WC, marital status, smoking status, alcohol consumption, self–rated health, complications(stroke, diabetes mellitus, coronary heart disease), diets, folate intake; Model 3 was adjusted for model 2 + components of the cardiometabolic risk factor (central obesity, impaired glucose metabolism, hypertension, low HDL–C and high triglycerides)

*BMI *Body mass index, *HDL–C *High–density lipoprotein cholesterol, *HR *Hazard ratio, *MASLD *Metabolic dysfunction–associated steatotic liver disease, *WC *Waist circumference

## Discussion

To our knowledge, this is the first study to link daily step counts and intensity to all-cause mortality in individuals with MASLD. Several key findings emerged from our results. First, even minimal levels of stepping activity (i.e., just above the 10th percentile of the exposure distribution, 3,800 steps per day for participants with MASLD) is associated with a roughly 30% reduction in all-cause mortality risk compared to those with 500 steps per day. Second, increasing daily steps is linked to a consistent decrease in mortality risk up to around 10,000 steps, beyond which the risk plateaus. Finally, we found no significant association between step intensity and all-cause mortality in the MASLD population.

While previous research has established the beneficial effects of increased step count on mortality in general and older populations, our study highlights the unique relevance of this relationship in MASLD [8, 23]. Several studies have explored the relationships between daily step counts and health outcomes, including cardiovascular disease, dementia, and all–cause mortality. For example, Cruz et al. [24] found that up to 10,000 steps per day may be linked to a decreased incidence of cardiovascular disease. Similarly, another study suggested that a number of steps fewer than 10,000 per day may be optimally associated with a lower risk of dementia [25]. However, a meta-analysis suggests that 8,000 steps per day may be the ideal threshold for reducing all-cause mortality risk, beyond which no further risk reduction is observed [26]. In this study, we found J-shaped nonlinear associations between daily step count and all-cause mortality. Notably, once the step count exceeds 10,000, the reduction in mortality risk seems to plateau, suggesting that higher step counts may not offer additional benefits for individuals with MASLD. Recent studies suggest that higher levels of exercise may increase the risks of adverse health outcomes. For example, DeFina et al. [27] demonstrated that strenuous joggers appeared to have higher mortality. Similarly, two studies [28, 29] found that higher levels of physical activity, such as marathon running, are linked to increased coronary artery calcification. These findings align with ours, emphasizing the need for step-count guidelines tailored to MASLD patients.

Studies of self-reported walking speed have found that higher walking speeds are associated with lower mortality risk [13]. However, the present study provides limited evidence that step intensity is associated with lower mortality, similar to findings from a study among older women [9]. One possible explanation for this inconsistency is that self-reported walking speed is subjective, while our study used an objective monitor to measure walking speed. After adjusting for age and gender confounders, peak 1 cadences showed that the highest intensity was not linked to reduced mortality, mainly due to individuals with lower step intensity being older. After adjusting for all included confounders factors, step intensity was no longer associated with mortality. Several factors may account for this finding. Firstly, adjustment for BMI and age is essential, as both are significant predictors of both physical activity levels and mortality in individuals with metabolic diseases [30, 31]. Particularly, age is a well–established factor influencing both the likelihood of engaging in physical activity and overall health outcomes. Older age is frequently associated with a decline in physical function, comorbid conditions, and increased mortality risk [32]. When adjusted for age and BMI, the independent effect of walking intensity on mortality may be attenuated, suggesting that the influence of physical activity on mortality could be mediated by these age–related factors. Additionally, including self-reported health status in our models may have further reduced the impact of walking intensity. Self–reported health serves as a proxy for various unmeasured factors, such as underlying comorbidities, physical function, and psychological health, all of which can influence both physical activity participation and mortality risk [33]. These factors may have confounded the relationship between walking intensity and survival, obscuring any potential direct effect of walking intensity on mortality. The cadences estimates (steps/min) used here should be interpreted with caution, as they reflect the steps accumulated per minute of accelerometer observation, not the steps taken during a full minute of walking [34]. Therefore, further studies using more accurate measures of walking speed are needed to confirm these findings.

This study has important practical implications for public health interventions targeting individuals with MASLD. Our findings suggest that achieving higher daily step counts, rather than focusing solely on step intensity, can significantly reduce the risk of all–cause mortality in this population. Given the simplicity and feasibility of increasing daily physical activity, healthcare providers may consider incorporating step count monitoring into routine care for MASLD patients to track progress and encourage sustained physical activity. By setting clear, measurable step goals, clinicians can improve patient compliance and potentially reduce long-term health risks associated with MASLD.

Future research should explore the combined effects of daily step counts and intensity on mortality in MASLD patients. Longitudinal studies using modern accelerometers could help clarify these associations, particularly in diverse MASLD populations with varying disease severities. Additionally, incorporating other lifestyle factors, such as diet and comorbid conditions, could provide a more comprehensive understanding of the impact of physical activity on mortality. Subgroup analyses based on disease stages (e.g., simple steatosis vs. steatohepatitis) could offer valuable insights into how physical activity interventions can be better tailored to enhance outcomes in MASLD patients.

Our study has several strengths. First, it was conducted using the NHANES database, which offers a population-based design and a large sample size, thus enhancing the robustness of our findings. Second, with a mean follow-up of 13.8 years, this study benefits from one of the longest durations exploring the association between step counts and mortality in MASLD patients.

However, the present study also has several limitations. First, the data presented are observational, precluding causal claims, even though we reanalyzed the data after excluding participants who experienced the event within the first two years of follow–up. Second, missing data and residual confounding may still affect the results. Third, the association between low step counts and mortality should be interpreted with caution due to the lack of data at low levels of exposure. Fourth, while data from a 7-day monitor are informative, they may not capture longer-term variations in step counts. Fifth, self-reported measures, although common in large-scale epidemiological studies, are prone to biases such as recall and social desirability bias. Sixth, cultural differences in reporting disease status and physical activity may influence results. Additionally, age-period-cohort (APC) effects may be present but were not assessed. The use of accelerometer data from 2005–2006, based on older technology, is another limitation. Finally, MASLD diagnosis relied on the FLI rather than liver biopsy.

## Conclusion

Our findings suggest that achieving 10,000 steps per day may be optimal to lower the risk of all-cause mortality among individuals with MASLD.

## Supplementary Information

Supplementary Material 1.

## Data Availability

The National Institutes of Health (NIH) managed data collection and baseline/mortality follow–up data management but did not participate in the study design, result analysis and interpretation, or manuscript drafting. The datasets presented in this study are available in online repositories. The names of the repository/repositories and accession number(s) are available in the link below: https://wwwn.cdc.gov/nchs/nhanes/Default.aspx.

## Abbreviations

MASLD: Metabolic dysfunction–associated steatotic liver disease
NHANES: National Health and Nutrition Examination Survey
FLI: Fatty Liver Index
BMI: Body mass index
WC: Waist circumference
GGT: Gamma glutamyl transpeptidase
HDL–C: High–density lipoprotein cholesterol
IRB: Institutional Review Board
peak 60 cadences: Peak 60–minute cadences
peak 30 cadences: Peak 30–minute cadences
peak 1 cadences: Peak 1–minute cadences
ORs: Odds ratios
