# Supplementary material for: Association of daily step counts and step intensity with mortality among US adults: a cross–sectional study of NHANES 2005–2006
Source: BMC Gastroenterol. 2025 Jan 20;25:21. doi: 10.1186/s12876-025-03606-7 (PMC11744998; doi:10.1186/s12876-025-03606-7)
Supplement: Supplementary file 1 — Supplementary Material 1. [file 12876_2025_3606_MOESM1_ESM.docx]

**Table 1. Multivariable Hazard Ratios of Steps per Day and All-Cause Mortality after adjusting for age and gender in peak 1-minute cadences**

**HR,** hazard ratio.

| **Variable** | **HR** | **P（Wald Test）** | **lower95** | **upper95** |
| --- | --- | --- | --- | --- |
| **Quartiles 1** | **Reference** |  |  |  |
| **Quartiles 2** | **0.6145** | **0.0081** | **0.4265** | **0.8604** |
| **Quartiles 3** | **0.4436** | **0.0134** | **0.2439** | **0.8248** |
| **Quartiles 4** | **0.5259** | **0.1329** | **0.2287** | **1.2433** |
| **Age** | **1.1005** | **< 0.001** | **1.0864** | **2.1148** |
| **Gender** | **0.70367** | **0.41431** | **0.5312** | **1.0322** |

**Table 2. Multivariable Hazard Ratios of Steps per Day and All-Cause Mortality after adjusting for all covariates in peak 1-minute cadences**

**HR,** hazard ratio**; BMI,** body mass index**; WC,** waist circumference; **HDL,** high-density lipoprotein**; TG,** triglycerides.

| **Variable** | **HR** | **P（Wald Test）** | **lower 95** | **upper95** |
| --- | --- | --- | --- | --- |
| **Quartiles 1** | **Reference** |  |  |  |
| **Quartiles 2** | **0.7763556** | **0.22239** | **0.51697** | **1.1659** |
| **Quartiles 3** | **0.8435326** | **0.4959** | **0.5169** | **1.3766** |
| **Quartiles 4** | **0.6524396** | **0.20968** | **0.33479** | **1.2715** |
| **Age** | **1.1000468** | **< 0.001** | **1.08016** | **1.1203** |
| **Gender** | **0.7335272** | **0.14753** | **0.48227** | **1.1157** |
| **Race (non-Hispanic whites)** | **Reference** |  |  |  |
| **Race (non-Hispanic blacks)** | **0.803881** | **0.32573** | **0.52013** | **1.2424** |
| **Race (Hispanic)** | **0.4833404** | **0.11132** | **0.37537** | **1.1484** |
| **Race (other Races)** | **1.6070088** | **0.2881** | **0.66975** | **3.8559** |
| **BMI** | **1.1951398** | **0.02881** | **1.04313** | **1.45** |
| **WC** | **1.0042235** | **0.73299** | **0.9802** | **1.0288** |
| **Smoking status (Never)** | **Reference** |  |  |  |
| **Smoking status (Former)** | **1.3864891** | **0.06465** | **0.98034** | **1.9609** |
| **Smoking status (Current)** | **0.8213093** | **0.32207** | **0.55627** | **1.2126** |
| **Poverty-income ratio (<1.3)** | **Reference** |  |  |  |
| **Poverty-income ratio (1.3–3.5)** | **0.5948301** | **0.23388** | **0.3681** | **1.1612** |
| **Poverty-income ratio (>3.5)** | **0.9816893** | **0.9613** | **0.46535** | **2.0709** |
| **Folate intake** | **0.9996995** | **0.56431** | **0.99868** | **1.0007** |
| **Low-carb and low-fat diets** | **1.0266474** | **0.87619** | **0.73747** | **1.4292** |
| **Diabetes Mellitus** | **0.8690673** | **0.48123** | **0.58811** | **1.2843** |
| **Education (Less than high school)** | **Reference** |  |  |  |
| **Education (High school)** | **0.8119758** | **0.08036** | **0.64291** | **1.0255** |
| **Education (More than high school)** | **0.7682758** | **0.17036** | **0.54675** | **1.2339** |
| **Marital status** | **1.2184829** | **0.25963** | **0.86418** | **1.718** |
| **Alcohol consumption** | **0.7767771** | **0.14495** | **0.55307** | **1.091** |
| **Coronary heart disease** | **0.8628051** | **0.55788** | **0.5267** | **1.4134** |
| **Stoke** | **1.2772132** | **0.43421** | **0.84708** | **1.9599** |
| **Self-rated health (Excellent)** | **Reference** |  |  |  |
| **Self-rated health (Very good)** | **1.2496325** | **0.02246** | **1.09026** | **1.9927** |
| **Self-rated health(good)** | **1.449625** | **0.03528** | **1.22859** | **2.1402** |
| **Self-rated health (Fair or poor)** | **1.6476087** | **0.02318** | **1.13913** | **2.2788** |
| **Obesity** | **0.2030226** | **0.14008** | **0.0443** | **1.1304** |
| **Low_HDL** | **1.2456253** | **0.24889** | **0.85753** | **1.8094** |
| **Hypertension** | **0.9025984** | **0.64336** | **0.58492** | **1.3928** |
| **High_TG** | **0.7506543** | **0.50176** | **0.32508** | **1.7333** |
| **Impaired glucose metabolism** | **0.6950717** | **0.08198** | **0.47686** | **1.9949** |

**Table 3. Multivariable Hazard Ratios of Steps per Day and All-Cause Mortality after adjusting for all covariates in peak 30-minute cadences**

**HR,** hazard ratio**; BMI,** body mass index**; WC,** waist circumference; **HDL,** high-density lipoprotein**; TG,** triglycerides.

| **Variable** | **HR** | **P（Wald Test）** | **lower95** | **upper95** |
| --- | --- | --- | --- | --- |
| **Quartiles 1** | **Reference** |  |  |  |
| **Quartiles 2** | **0.713906** | **0.1286** | **0.46227** | **1.1025** |
| **Quartiles 3** | **0.7348786** | **0.2765** | **0.42195** | **1.2799** |
| **Quartiles 4** | **0.653277** | **0.1948** | **0.34322** | **1.2434** |
| **Age** | **1.0980417** | **< 0.001** | **1.07788** | **1.1186** |
| **Gender** | **0.7162692** | **0.1204** | **0.47009** | **1.0914** |
| **Race (non-Hispanic whites)** | **Reference** |  |  |  |
| **Race (non-Hispanic blacks)** | **0.8109814** | **0.3435** | **0.52573** | **1.251** |
| **Race (Hispanic)** | **0.488472** | **0.2127** | **0.2781** | **1.158** |
| **Race (other Races)** | **1.6496704** | **0.2622** | **0.68768** | **3.9574** |
| **BMI** | **1.165522** | **0.0121** | **1.0943** | **1.451** |
| **WC** | **1.0034655** | **0.7819** | **0.97919** | **1.0283** |
| **Smoking status (Never)** | **Reference** |  |  |  |
| **Smoking status (Former)** | **1.009636** | **0.3332** | **0.96917** | **1.9427** |
| **Smoking status (Current)** | **0.8512841** | **0.4191** | **0.57603** | **1.2581** |
| **Poverty-income ratio (<1.3)** | **Reference** |  |  |  |
| **Poverty-income ratio91.3–3.5)** | **0.6277284** | **0.0597** | **0.38663** | **1.0192** |
| **Poverty-income ratio (>3.5)** | **1.039729** | **0.9187** | **0.49226** | **2.1961** |
| **Folate intake** | **0.9997262** | **0.599** | **0.99871** | **1.0007** |
| **Low-carb and low-fat diets** | **1.0314706** | **0.855** | **0.7398** | **1.4381** |
| **Diabetes Mellitus** | **0.889138** | **0.5569** | **0.60076** | **1.316** |
| **Education (Less than high school)** | **Reference** |  |  |  |
| **Education (High school)** | **0.8180286** | **0.0902** | **0.64844** | **1.032** |
| **Education (More than high school)** | **0.5621852** | **0.1625** | **0.36814** | **1.1056** |
| **Marital status** | **1.187159** | **0.3252** | **0.84345** | **1.6709** |
| **Alcohol consumption** | **0.7747814** | **0.1402** | **0.55197** | **1.0875** |
| **Coronary heart disease** | **0.8443019** | **0.5031** | **0.5145** | **1.3855** |
| **Stoke** | **0.5847977** | **0.0785** | **0.35182** | **1.0721** |
| **Self-rated health (Excellent)** | **Reference** |  |  |  |
| **Self-rated health (Very good)** | **1.1434188** | **0.0299** | **1.09958** | **2.1793** |
| **Self-rated health(good)** | **1.320918** | **0.0175** | **1.19111** | **2.3518** |
| **Self-rated health (Fair or poor)** | **1.4213571** | **0.0102** | **1.12651** | **2.2246** |
| **Obesity** | **0.7835764** | **0.1385** | **0.53972** | **1.8485** |
| **Low_HDL** | **1.2215429** | **0.8186** | **0.84294** | **1.7701** |
| **Hypertension** | **0.8995709** | **0.633** | **0.5826** | **1.389** |
| **High_TG** | **0.806652** | **0.6147** | **0.34943** | **1.8621** |
| **Impaired glucose metabolism** | **0.6873098** | **0.0738** | **0.45566** | **1.0367** |

**Table 4. Multivariable Hazard Ratios of Steps per Day and All-Cause Mortality after adjusting for all covariates in peak 1-minute cadences**

**HR,** hazard ratio**; BMI,** body mass index**; WC,** waist circumference; **HDL,** high-density lipoprotein**; TG,** triglycerides.

| **Variable** | **HR** | **P（Wald Test）** | **lower95** | **upper95** |
| --- | --- | --- | --- | --- |
| **Quartiles 1** | **Reference** |  |  |  |
| **Quartiles 2** | **0.74583** | **0.17068** | **0.49027** | **1.1346** |
| **Quartiles 3** | **0.770693** | **0.39126** | **0.42489** | **1.3979** |
| **Quartiles 4** | **0.605956** | **0.14376** | **0.30957** | **1.1861** |
| **Age** | **1.098711** | **< 0.001** | **1.07828** | **1.1195** |
| **Gender** | **0.726891** | **0.1371** | **0.47735** | **1.1069** |
| **Race (Non-Hispanic White)** | **Reference** |  |  |  |
| **Race (Non-Hispanic Black)** | **0.806444** | **0.33413** | **0.52118** | **1.2479** |
| **Race (Hispanic)** | **0.489318** | **0.21279** | **0.27875** | **1.1589** |
| **Race (Other Races)** | **1.675004** | **0.24749** | **0.6988** | **4.0149** |
| **BMI** | **1.295946** | **0.02272** | **1.09436** | **1.8512** |
| **WC** | **1.003279** | **0.79261** | **0.97909** | **1.0281** |
| **Smoking status (Never)** | **Reference** |  |  |  |
| **Smoking status (Former)** | **1.367867** | **0.07801** | **0.96548** | **1.938** |
| **Smoking status (Current)** | **0.862408** | **0.46052** | **0.58207** | **1.2778** |
| **Poverty-income ratio (<1.3)** | **Reference** |  |  |  |
| **Poverty-income ratio91.3–3.5)** | **0.631659** | **0.06466** | **0.388** | **1.0283** |
| **Poverty-income ratio (>3.5)** | **1.058188** | **0.88261** | **0.4995** | **2.2418** |
| **Folate intake** | **0.999759** | **0.64361** | **0.99874** | **1.0008** |
| **Low-carb and low-fat diets** | **1.01461** | **0.93149** | **0.72895** | **1.4122** |
| **Diabetes Mellitus** | **0.889685** | **0.55898** | **0.60114** | **1.3167** |
| **Education (Less than high school)** | **Reference** |  |  |  |
| **Education (High school)** | **0.816206** | **0.08735** | **0.64667** | **1.0302** |
| **Education (More than high school)** | **0.7536** | **0.1527** | **0.468283** | **1.3958** |
| **Marital status** | **1.164124** | **0.38644** | **0.82536** | **1.6419** |
| **Alcohol consumption** | **0.781964** | **0.15547** | **0.55694** | **1.0979** |
| **Coronary heart disease** | **0.842721** | **0.49825** | **0.51358** | **1.3828** |
| **Stoke** | **0.69617** | **0.73972** | **0.35005** | **1.2699** |
| **Self-rated health (Excellent)** | **Reference** |  |  |  |
| **Self-rated health (Very good)** | **1.143562** | **0.01569** | **1.01998** | **2.0803** |
| **Self-rated health(good)** | **1.335989** | **0.03016** | **1.19783** | **2.0856** |
| **Self-rated health (Fair or poor)** | **1.508543** | **0.02507** | **1.31987** | **2.1873** |
| **Obesity** | **0.675985** | **0.32615** | **0.33807** | **1.8136** |
| **Low_HDL** | **1.216895** | **0.29981** | **0.34657** | **1.8519** |
| **Hypertension** | **0.874715** | **0.54608** | **0.56639** | **1.3509** |
| **High_TG** | **0.801139** | **0.60403** | **0.34657** | **1.8519** |
| **Impaired glucose metabolism** | **1.066283** | **0.86094** | **0.52002** | **2.1864** |
